# A Large-Scale Complex Haploinsufficiency-Based Genetic Interaction Screen in *Candida albicans*: Analysis of the RAM Network during Morphogenesis

**DOI:** 10.1371/journal.pgen.1002058

**Published:** 2011-04-28

**Authors:** Nike Bharucha, Yeissa Chabrier-Roselló, Tao Xu, Cole Johnson, Sarah Sobczynski, Qingxuan Song, Craig J. Dobry, Matthew J. Eckwahl, Christopher P. Anderson, Andrew J. Benjamin, Anuj Kumar, Damian J. Krysan

**Affiliations:** 1 Department of Molecular, Cellular, and Developmental Biology, University of Michigan, Ann Arbor, Michigan, United States of America; 2 Department of Pediatrics, University of Rochester School of Medicine and Dentistry, Rochester, New York, United States of America; 3 Department of Microbiology/Immunology, University of Rochester School of Medicine and Dentistry, Rochester, New York, United States of America; Stanford University School of Medicine, United States of America

## Abstract

The morphogenetic transition between yeast and filamentous forms of the human fungal pathogen *Candida albicans* is regulated by a variety of signaling pathways. How these pathways interact to orchestrate morphogenesis, however, has not been as well characterized. To address this question and to identify genes that interact with the Regulation of Ace2 and Morphogenesis (RAM) pathway during filamentation, we report the first large-scale genetic interaction screen in *C. albicans*. Our strategy for this screen was based on the concept of complex haploinsufficiency (CHI). A heterozygous mutant of *CBK1* (*cbk1*Δ/*CBK1*), a key RAM pathway protein kinase, was subjected to transposon-mediated, insertional mutagenesis. The resulting double heterozygous mutants (6,528 independent strains) were screened for decreased filamentation on Spider Medium (SM). From the 441 mutants showing altered filamentation, 139 transposon insertion sites were sequenced, yielding 41 unique *CBK1*-interacting genes. This gene set was enriched in transcriptional targets of Ace2 and, strikingly, the cAMP-dependent protein kinase A (PKA) pathway, suggesting an interaction between these two pathways. Further analysis indicates that the RAM and PKA pathways co-regulate a common set of genes during morphogenesis and that hyper-activation of the PKA pathway may compensate for loss of RAM pathway function. Our data also indicate that the PKA–regulated transcription factor Efg1 primarily localizes to yeast phase cells while the RAM–pathway regulated transcription factor Ace2 localizes to daughter nuclei of filamentous cells, suggesting that Efg1 and Ace2 regulate a common set of genes at separate stages of morphogenesis. Taken together, our observations indicate that CHI–based screening is a useful approach to genetic interaction analysis in *C. albicans* and support a model in which these two pathways regulate a common set of genes at different stages of filamentation.

## Author Summary

*Candida albicans* is the most common cause of fungal infections in humans. As a diploid yeast without a classical sexual cycle, many genetic approaches developed for large-scale genetic interaction studies in the model yeast *Saccharomyces cerevisiae* cannot be applied to *C. albicans*. Genetic interaction studies have proven to be powerful genetic tools for the analysis of complex biological processes. Here, we demonstrate that libraries of *C. albicans* strains containing heterozygous mutations in two different genes can be generated and used to study genetic interactions in *C. albicans* on a large scale. Double heterozygous mutants that show more severe phenotypes than strains with single heterozygous mutations are indicative of genetic interactions through a phenomenon referred to as complex haploinsufficiency (CHI). We applied this approach to the study of the RAM (Regulation of Ace2 and Morphogenesis) signaling network during the morphogenetic transition of *C. albicans* from yeast to filamentous growth. Among the genes that interacted with *CBK1*, the key signaling kinase of the RAM pathway, were transcriptional targets of the RAM pathway and the protein kinase A pathway. Further analysis supports a model in which these two pathways co-regulate a common set of genes at different stages of filamentation.

## Introduction

*Candida albicans* is a member of the resident flora of the gastrointestinal tract and is the most common fungal pathogen in humans. The most severe manifestations of candidiasis occur in immunocompromised patients and include debilitating mucosal disease such as oropharyngeal candidiasis as well as life-threatening disseminated infections of the bloodstream and major organ systems [1]. Animal studies have shown that the pathogenic potential of *C. albicans* is associated with its ability to transition between three morphological states: yeast, pseudohyphae, and hyphae [2], [3]. Further insights into the contributions of the different morphotypes to pathogenesis have emerged from elegant studies with *C. albicans* strains that allow the conditional induction of filamentation *in vivo* [4]. For example, *C. albicans* genetically restricted to the yeast form by constitutive expression of *NRG1* are able to establish infection in mice but no disease results until the expression of *NRG1* is repressed and the organism is able to form filaments.

The relationship between morphogenesis and virulence in *C. albicans* is, however, not a simple one. Many mutants that are unable to undergo morphogenesis also display other phenotypes. For example, many transcription factors that are required for morphogenesis regulate a host of other genes and display pleiomorphic phenotypes. The complicated nature of the relationship between morphogenesis has been further highlighted by the elegant study recently reported by Noble *et al.* [5]. Noble *et al.* generated a bar-coded collection of homozygous deletion mutants and used it in a signature-tagged mutagenesis study of infectivity in a mouse model [5]. Mutants with defects in morphogenesis were more likely to have decreased infectivity; however, a significant portion of mutants with severe morphogenesis defects retained the ability to cause infection. It is important to note that Noble *et al.* assayed for infection and not for disease. Thus, their results are not necessarily in conflict with studies discussed above that indicate that morphogenesis is required for disease progression in animal models [4]. Furthermore, their work serves to highlight the fact that additional studies will be required to fully understand the complex relationship between morphogenesis and pathogenesis in *C. albicans*.

Given the close association of morphogenesis with *C. albicans* pathogenesis, the genetic and cell biologic analysis of this process has been the subject of intensive study [6]. Consequently, many genes have been shown to affect filamentation, and, correspondingly, a number of regulatory pathways have been shown to play a role in the orchestration of the morphogenetic program in *C. albicans* [7]. The *PKA*, *CPH1*, *HOG1*, *RIM101*, *CHK1*, and *CBK1*/RAM pathways are among those that regulate morphogenesis under a variety of conditions [6], [7]. Although much remains to be learned about how individual pathways and genes contribute to morphogenesis, an important question that has not been extensively studied is how these various pathways interact to regulate morphogenesis.

In the model yeast *S. cerevisiae*, relationships between regulatory pathways can be readily characterized using recently developed systematic, genome-wide genetic interaction strategies [8]–[10]. These approaches have yielded a wealth of information regarding the mechanisms through which cells regulate complex biological processes [11]. However, because *C. albicans* is diploid and lacks a classical meiotic cycle, the mating-based genetic strategies used to create genome-wide libraries of double mutant strains in *S. cerevisiae* are not applicable. Consequently, genetic interaction studies in *C. albicans* have been limited to gene-by-gene analyses. Despite these limitations, such studies have proven quite informative and suggest that large scale interaction studies could represent a powerful approach to studying regulatory networks in *C. albicans*. For example, Braun *et al.* carried out a thorough, systematic epistasis analysis of three transcriptional regulators (*EFG1*, *TUP1* and *CPH1*) and showed that each played a distinct role in the regulation of filamentation [12].

Recent advances in the genetic analysis of *C. albicans* have greatly facilitated the development of innovative approaches to the study of this important human pathogen [13]. Among these important developments is the application of transposon-based mutagenesis strategies [14] to the creation and study of large-scale libraries of heterozygous [15], and homozygous [17], [18] *C. albicans* mutants. Similarly, large collections of homozygous null [19] and conditional mutants [20] have been created in a targeted manner and analyzed for a variety of phenotypes including morphogenesis, virulence and drug susceptibility. To our knowledge, one area that has not been explored is the development of approaches to large-scale synthetic genetic interaction analysis in *C. albicans*.

Here, we describe the first large-scale synthetic genetic interaction screen in *C. albicans*. Our strategy builds on pioneering yeast genetics approaches developed in both *S. cerevisiae* and *C. albicans* and is based on the concept of complex haploinsufficiency (CHI). CHI is a special case of a genetic phenomenon referred to as unlinked non-complementation in the context of yeast genetics and as dominant enhancers or dominant modifiers when applied to *Drosophila* [21]. Unlinked non-complementation occurs when a cross between two haploid strains containing single recessive mutations located in separate loci results in a diploid strain (complex heterozygote) that retains the phenotype of a parental strain. In yeast, the construction of such mutants was used to great advantage in the genetic analysis of cytoskeletal genes such as tubulin [22] and actin [23]. CHI, which is a special case of unlinked non-complementation, occurs when strains containing heterozygous mutations at two separate loci display a more severe phenotype than strains that contain heterozygous mutations at the single loci alone [21]. In essence, CHI can also be called synthetic haploinsufficiency. Recently, a genome-wide CHI-based strategy was developed in *S. cerevisiae* and successfully used to create a genetic interaction network for the essential gene, *ACT1* [21].

As described in the seminal work of Uhl *et al.* [15], large scale haploinsufficiency-based screening was first applied to *C. albicans* in the transposon-mediated, insertional mutagenesis analysis of filamentation and, thus, haploinsufficiency-based screening has excellent precedence in this system. Whereas Uhl *et al.* carried out their haploinsufficiency screen starting with a “wild type” strain [15], we reasoned that transposon mutagenesis of a parental strain containing a heterozygous mutation at a locus of interest would represent an expedient approach to the generation of a large library of complex heterozygotes that could then be the basis of a CHI screen for genes that interact with the parental mutant.

In principle, CHI-based screening has a number of attractive features. First, CHI allows one to identify genes that function within the pathway affected by the parental or query mutation including upstream and downstream components of the pathway, transcriptional outputs of the pathway, and substrates of pathway enzymes. Second, CHI-based screening can also identify genes or pathways that function in parallel with the query pathway and, therefore, allow one to identify pathways that co-regulate a given process. Third, CHI is ideal for the study of essential genes because only heterozygous mutations are generated.

**Figure 1:**
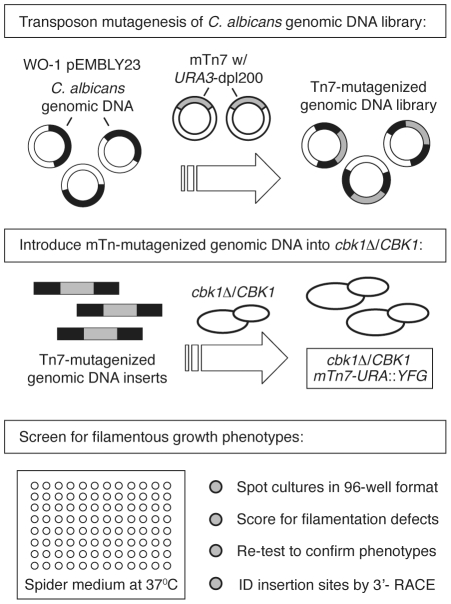
Schematic of screening strategy. *In vitro* mutagenesis of *C. albicans* genomic library WO-1 using a Tn7-based transposon containing the *CaURA3-dpl200* auxotrophic marker [29] yielded a library of plasmids from which genomic inserts were released by restriction endonuclease digestion and transformed into a *cbk1*Δ/*CBK1* heterozygote strain. The resulting library was screened on SM for altered filamentation relative to the parental strain.

We developed a CHI-based screening strategy (Figure 1) and applied it to the identification of genes that interact with the RAM signaling network during *C. albicans* filamentation [24]–[27]. The RAM network has been extensively studied in *S. cerevisiae* [28] and is required for a variety of cellular processes in both *S. cerevisiae* and *C. albicans* including polarity, cell wall synthesis, cell separation and filamentous growth. Cbk1 is the key serine/threonine protein kinase [24], [27] that mediates many of the functions of the RAM network through its regulation of the transcription factor Ace2 [24], [27]. RAM pathway mutants in *C. albicans* show two distinct filamentation phenotypes: *CBK1* null mutants are unable to form filaments on Spider Medium (SM) or serum-containing medium [24], [27] while *ACE2* null mutants are constitutively pseudohyphal and form true hyphae on serum [25]. Although our understanding of the RAM network in *C. albicans* has increased in recent years [24]–[27], many questions remain, including: how does it interact with the many other regulatory pathways during morphogenesis and what genes and proteins are regulated by Cbk1 and/or its downstream transcription factor Ace2?

Through this novel application of a CHI-based screening strategy, we have identified RAM/Ace2 transcriptional targets and generated genetic evidence for an interaction between the RAM and PKA pathways during morphogenesis. Follow-up studies of the screening results further suggest that a balance between RAM and PKA-pathway activity is required for cells to establish a normal distribution of morphotypes during nutrient-induced filamentation. Taken together with previous work on these two pathways, our observations support a model where PKA-regulated transcriptional activity is most important in the transcription of RAM/PKA co-regulated genes early in morphogenesis, while the RAM pathway is more important as daughter nuclei accumulate within the hyphal structure.

## Results

### Construction of the insertional library and CHI screening strategy

An outline of the CHI-based screening strategy is presented in Figure 1. In preparation for the CHI screen, we first constructed a transposon suitable for large-scale insertional mutagenesis in *C. albicans*. To enable efficient mutagenesis with limited transposition bias, we generated a donor plasmid derived from the bacterial element Tn*7*. The Tn*7* system has been used extensively for *in vitro* mutagenesis [29], [30] with low reported insertion site specificity [31]. For purposes of this screen, the Tn*7* element was modified to contain a recyclable allele of the *CaURA3* gene; specifically, we inserted the *URA3-dpl200* allele into Tn*7* sequence encoded in the donor plasmid pGPS3. The *URA3-dpl200* allele was designed by Wilson *et al.* [32] to allow recombinational excision of the *URA3* gene under counter-selection with 5-fluoro-orotic acid (5-FOA). Subsequently, we performed *in vitro* mutagenesis of the genomic library pEMBLY23 (Materials and Methods) derived from *C.albicans* strain WO-1. Non-specific Tn*7* transposition was achieved using the TnsA, TnsB, and TnsC* proteins paired with the TnsAB transposase and appropriate cofactors [29]. The genomic library was mutagenized to yield an estimated 20,000 independent insertions. The resulting insertional library was recovered in *E. coli*, and genomic DNA inserts were released by enzyme digestion for introduction into the *C. albicans* Ura- parental strain, *cbk1*Δ/*CBK1* (CAMM292, see Table S1 for strain table). By homologous recombination, the mutagenized genomic DNA fragment will replace its native chromosomal locus, thereby generating a heterozygous insertion mutant in the parent *cbk1*Δ/*CBK1* strain. DNA transformations were performed nine times, yielding a total of 6528 independent Ura+ transformants. The *C. albicans* double heterozygotes were isolated and screened for decreased filamentation as follows.

### CHI screening of the *cbk1*Δ/*CBK1* mutant

**Figure 2:**
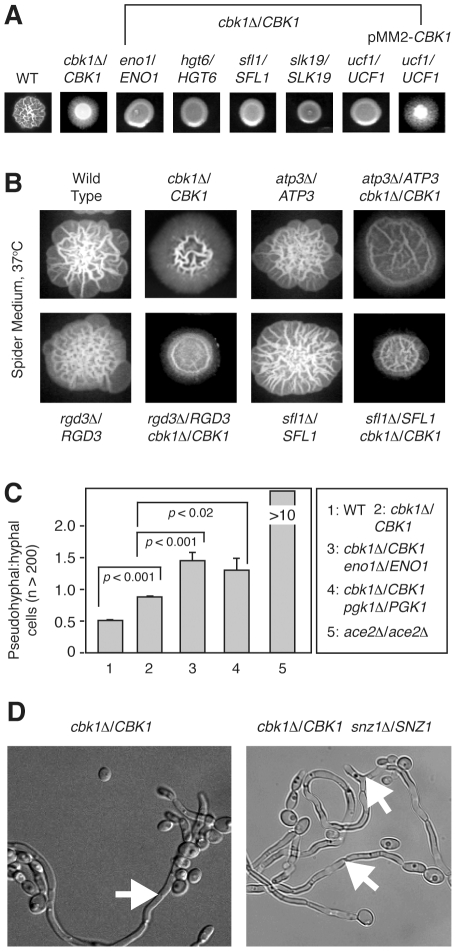
CHI–based screening identifies synthetic genetic interactions with *CBK1* during morphogenesis. (A) Examples of primary screening data for complex heterozygotes showing synthetic genetic interactions with *CBK1*; each strain was spotted on SM and incubated at 37°C for 3 days. Mutants with decreased peripheral invasion and decreased central wrinkling were selected. Representative positive scoring mutants from the primary screen are shown. An example of a strain complemented by re-integration of plasmid-borne *CBK1* is shown. (B) Representative examples of independently constructed complex heterozygote strains showing complex haploinsufficient genetic interactions with *cbk1*Δ. (C) The ratio of pseudohyphal∶hyphal cells for the indicated strains was determined by light microscopy after 3 hours incubation in liquid SM at 37°C. The bars indicate mean values of two-three independent replicates of at least 100 cells. Error bars indicate standard deviation. Brackets indicate the results of Student's *t* test evaluation of differences between the indicated mutants; *p*<0.05 indicates a statistically significant difference. (D) Micrograph of filaments isolated from colonies of the parental *cbk1*Δ/*CBK1* strain and the complex heterozygote *cbk1*Δ/*CBK1 snz1*Δ/*SNZ1*. Arrowheads indicate areas of hyphal-like morphology in the *cbk1*Δ/*CBK1* mutant and pseudohyphae-like morphology in the *cbk1*Δ/*CBK1 snz1*Δ/*SNZ1* double mutant.

The *cbk1*Δ/*CBK1* mutant was originally studied in *C. albicans* by McNemar and Fonzi [24] and was found to be haploinsufficient with respect to filamentation on a variety of media. Uhl *et al.* also isolated a heterozygous *cbk1* insertion mutant in their haploinsufficiency screen [15]. As shown in Figure 2A, *cbk1*Δ/*CBK1* colonies show a decreased area of central wrinkling and a more prominent ring of peripheral filamentation on SM at 37°C. The haploinsufficiency of this parental strain was advantageous for two reasons. First, it provided increased sensitivity in that the strain was already deficient for filamentation. Second, it could also improve specificity because weak phenotypes of non-interacting, transposon-derived mutants would not be apparent due to masking by the *cbk1*Δ/*CBK1* phenotype.

As described above [24], [25], [27], RAM pathway mutants show two distinct phenotypes depending on the conditions used to induce filamentation, but both phenotypes are apparent on solid Spider Medium (SM). In order to identify mutations that potentially interacted with both general functions of the pathway, we, therefore, screened for decreased filamentation on SM at 37°C. All subsequent experiments were conducted under these conditions unless otherwise indicated.

The library of 6528 complex heterozygous mutants was spotted in 96-well format and scored for decreased peripheral invasion and altered colony wrinkling relative to a Ura+ derivative of the parental *cbk1*Δ/*CBK1* strain (11, Figure 2A). Clones showing both phenotypes were re-tested using two independent colonies. A total of 441 complex heterozygous mutants with decreased peripheral invasion and altered colony wrinkling were re-confirmed on both SM and SM containing uridine to control for positional effects of the *URA3* gene (Figure 2A). We specifically selected mutants with *decreased* zones of peripheral filamentation and *less* pronounced central wrinkling relative to the parental strain (Figure 2A and 2B). All mutants showed some degree of peripheral filamentation. The most common composite phenotype indicated a small zone of peripheral agar invasion with a broad region of moderate wrinkling (Figure 2B).

**Figure 3:**
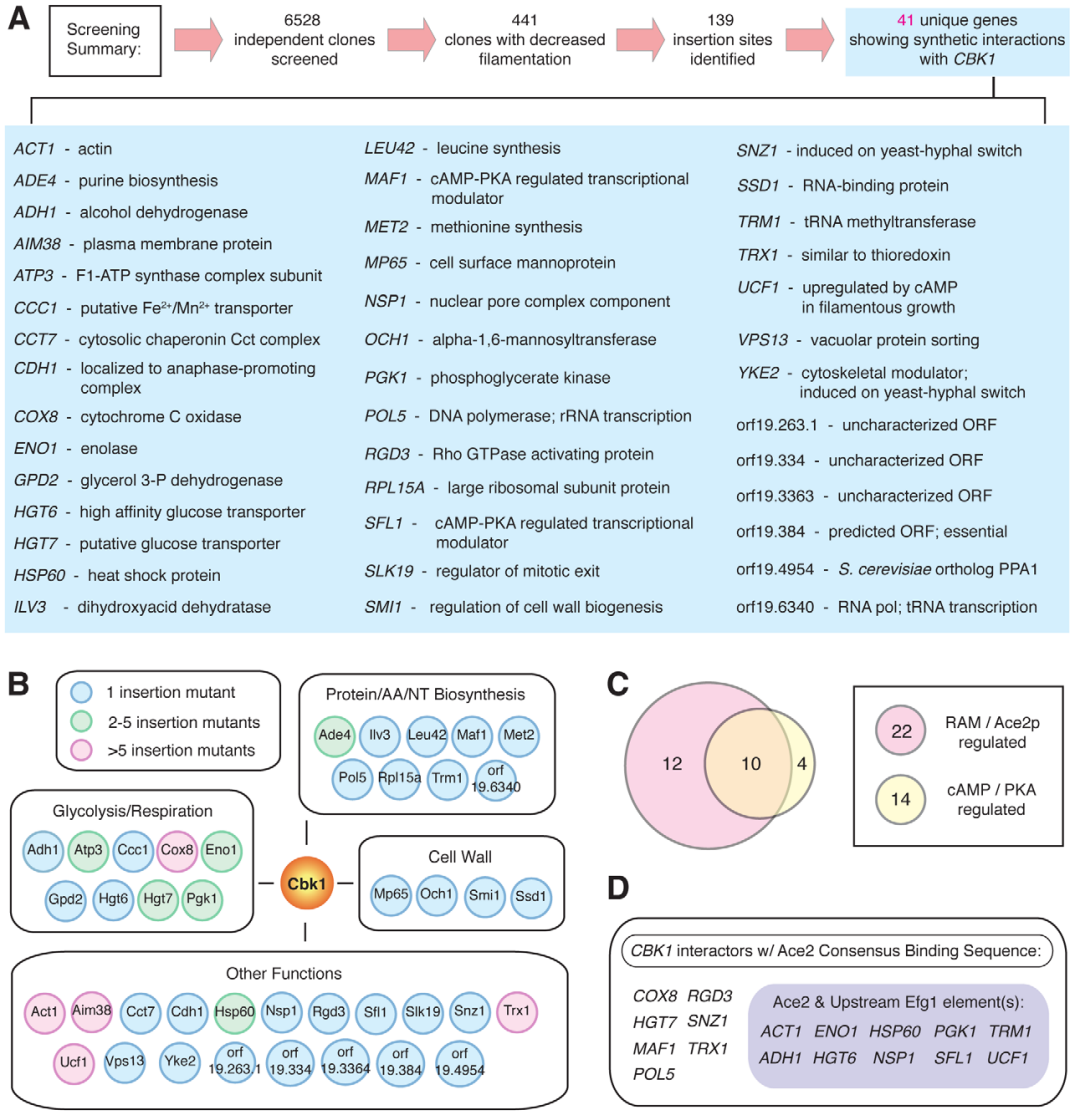
Summary and bioinformatic analysis of screening data. (A) Summary of screening results and list of *CBK1*-interacting genes. (B) List of *CBK1*-synthetic genetic interactions during morphogenesis grouped according to three most common GO terms. Colors indicate the number of times each insertion was isolated. (C) Venn diagram depicting the number of genes putatively co-regulated by the RAM and PKA pathways. (D) List of *CBK1*-interacting genes with Ace2 and both Ace2/Efg1 consensus binding sites within the region 1000 bp upstream of the start codon.

The transposon insertion sites for approximately one-third of the mutants (139 strains) showing potential synthetic genetic interactions were identified using a 3′-RACE/sequencing approach (see Materials and Methods), yielding 42 unique transposon-derived mutations as putative *CBK1*-interactors. Since 8 insertion sites were identified in at least 5 separate clones (Figure 3A and 3B), the screen appeared to be saturated to the limits of the library and the mutagenesis technique. Therefore, we did not sequence the remaining two-thirds of the mutants and focused on evaluating the initial set of 42 mutants. It is, however, important to note that the screen itself is unlikely to be saturated for all possible *CBK1* interactors, as the library almost certainly did not contain insertions in all predicted *C. albicans* genes.

The *URA3* marker was recycled from the heterozygotes by 5-FOA mediated recombinational excision [32]. Following phenotypic re-testing to confirm that homozygosis was not responsible for curing the *URA3* marker, *CBK1* was re-integrated at its chromosomal position using plasmid pMM4 [24]. The phenotypes of 41 of 42 candidate CHI strains were modified by re-integration of *CBK1* (Figure 2A), indicating that the observed phenotypes were dependent on the *cbk1* mutation and were likely due to a synthetic genetic interaction between *cbk1*Δ/*CBK1* and the transposon insertion. The high percentage of *CBK1*-dependent phenotypes may be due to the fact that the parental *cbk1*Δ heterozygote is itself haploinsufficient on SM and most non-interacting insertion mutations that are themselves haploinsufficient do not have sufficiently strong phenotypes to appreciably change the phenotype of the double heterozygote relative to the parental strain. To confirm these interactions further, a subset of ten complex heterozygous mutants was independently constructed from CAMM-292 by single gene-replacement [33]. All ten double mutants recapitulated phenotypes displayed by the transposon-derived mutants and showed distinct phenotypes relative to strains with single deletions of the interacting genes. Representative images from this analysis are shown in Figure 2B.

To further characterize the morphologies of the mutants, we determined the proportion of yeast, pseudohyphae and hyphae after 3 hours induction in liquid SM at 37°C. The interacting mutants consistently showed an increased proportion of pseudohyphal cells relative to wild type and *cbk1*Δ/*CBK1* strains (Figure 2C). Similarly, examination of cells scraped from SM plates showed that the filaments of double mutants had constricted septal areas characteristic of pseudohyphae (Figure 2D). Importantly, all of the mutants were indistinguishable from wild type and the parental strain when serum was used as the inducer of filamentation (data not shown). Since *ace2*Δ/Δ mutants also show decreased peripheral invasion, decreased central wrinkling, increased levels of pseudohyphae, and normal filamentation on serum (25), we conclude that the majority of the *CBK1*-interacting genes isolated in the screen appear to affect the Ace2-dependent functions of the RAM pathway.

### The set of *CBK1*-interactors contains genes related to Ace2 function

Literature analysis of the set of *CBK1*-interactoring genes revealed that approximately one-half are involved in glycolysis/respiration, biosynthesis, or cell wall metabolism (Figure 3B), cell processes consistent with established functions of the RAM pathway [24]–[28]. An important interactor in terms of validating the screen is *SSD1* because it is a likely Cbk1 substrate in *S. cerevisiae* [34], displaying well-characterized genetic interactions with *CBK1* in both *S. cerevisiae* [35] and *C. albicans* [27]. Comparison of our dataset with that generated by the haploinsufficiency screen of Uhl *et al.* revealed no overlap [15]. As discussed above, we suspect that this lack of overlap is also related to the fact that our parental strain is haploinsufficient for filamentation and, thus, non-interacting transposon-derived mutations causing simple haploinsufficiency were, in effect, masked by the phenotype of the parental strain.

In principle, the Ace2-deficient phenotypes displayed by the double heterozygous mutants could result from mutations that interfere with the activation of Ace2 or from mutations that affect a key transcriptional target of Ace2. We isolated three mutants that could cause a CHI-interaction with *CBK1* through the former mechanism. First, we isolated orthologs of two genes that regulate mitotic exit in *S. cerevisiae*, *CDH1* [36] and *SLK19* [37]. Ace2 is well known to localize to the nuclei of daughter cells in both *S. cerevisiae* [38] and *C. albicans* [25], [39]. Since Cdh1 and Slk19 regulate mitotic exit, the point in the cell cycle when Ace2 localizes to the nuclei [38], we suggest that disruption of mitotic exit through the loss of these proteins may further decrease the overall activity of Ace2. In addition, *NSP1*, a key component of the nuclear import machinery, was isolated. Studies in *S. cerevisiae* [40] have indicated that decreased *NSP1* gene dosage leads to inhibition of nuclear import, and it seems plausible that a strain lacking an allele of *NSP1* could have decreased nuclear import of Ace2 which would further decrease the overall Ace2-mediated transcriptional activity of the *cbk1*Δ/*CBK1* mutant.

**Figure 4:**
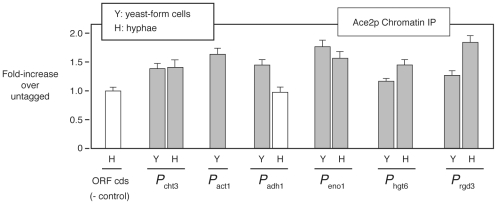
The set of *CBK1*-interacting genes includes transcriptional targets of Ace2. The binding of Ace2-TAP to the promoter regions of 5 *CBK1*-interacting genes was assessed by ChIP in yeast and hyphae-phase cells (SM, 3 h, 37°C) containing a TAP-tag fused to the C-terminus of Ace2. Bars indicate the ratio of promoter DNA (determined by PCR) in tagged extracts relative to un-tagged extracts (error bars indicate SD of three replicates). Grey bars show promoters with increased abundance in tagged extracts, suggesting they are bound by Ace2. *P*_cht3_, a known target of Ace2 [13], [21], and primers to a coding sequence (ORF cds) serve as positive and negative controls. A persistent contaminating band prevented accurate assessment of *ACT1* in hyphae.

The larger class of *CBK1*-interacting mutants that relate to Ace2 function is the set of genes that appear to be part of the transcriptional output of the RAM pathway (Figure 3C and 3D). To identify such genes in our data set, we searched the promoter regions of *CBK1*-interactors and found 22 genes that contain a *C. albicans* Ace2-consensus binding sequence [MMCCASC, 26]. Of these genes, 11 have been shown to display decreased expression in *ace2*Δ/Δ mutants during hyphal induction as reported in a recent transcriptional profiling study [26]. To further confirm that our screen identified genes regulated by Ace2, we examined the binding of Ace2 to the promoters of 5 *CBK1*-interactors with consensus binding sites (*ACT1*, *ADH1*, *ENO1*, *HGT6*, & *RGD3*) during both yeast and hypha-phase growth by chromatin immunoprecipitation (ChIP). Consistent with ChIP data for Ace2 reported by Wang *et al.* [39], the absolute enrichment was relatively low, most likely due to its cell cycle regulation and our non-synchronous experiments (Figure 4). Nevertheless, all five promoters were bound by Ace2 at levels comparable to those observed for the well-established Ace2 target *CHT3* and to those reported by Wang et al. [39] during yeast growth. In addition, three promoters were bound in hyphal phase (Figure 4). Taken together, the presence of Ace2 binding sites, the transcriptional profiling data, and ChIP data support the notion that many of the *CBK1*-interacting genes are transcriptional targets of Ace2.

### The CHI screen of *cbk1*Δ/*CBK1* reveals potential interaction between the RAM and PKA pathways during morphogenesis

Comparison of the set of *CBK1*-interactors with data from a variety of transcriptional profiles of *C. albicans* morphogenesis indicated that a substantial subset of *CBK1*-interactors (14 interactors, 34%) are regulated by the cAMP/PKA pathway through the transcription factor Efg1 [41]. Indeed, 10 *CBK1*-interactors contain consensus binding sites for both Ace2 and Efg1 (Figure 3C and 3D), suggesting that these two transcription factors may regulate a common set of genes. Further supporting this notion are previous studies indicating that both Ace2 and Efg1 induce glycolytic genes and repress genes involved in oxidative respiration [26], [41]. Indeed, we searched the *C. albicans* genome and found that the promoters of 384 genes contain consensus binding sites for both Ace2 and Efg1 (Table S2). Consistent with previous studies of the two pathways, the set of putatively co-regulated genes is enriched for genes contributing to glycolysis, biosynthesis, and cellular stress responses. Recently, Wang *et al.* have also shown that the promoters of Ace2-regulated cell wall and cell separation genes are bound by both Efg1 and Ace2 during morphogenesis [39]. Taken together our genetic data strongly support the notion that genes regulated by the PKA pathway may also be important components of the transcriptional output of the RAM pathway during morphogenesis.

In addition to transcriptional targets of the PKA pathway, three other *CBK1* interactors (*MAF1*, *SLF1* & *ACT1*) have connections to the PKA pathway (Figure 3A). *MAF1* and *SFL1* are both orthologs of PKA-regulated transcriptional regulators in *S. cerevisiae* [42], [43], suggesting that proper PKA-mediated transcriptional control is important in the absence of full RAM pathway activity. Further suggesting that the activity of the PKA pathway is important in RAM pathway mutants, we isolated *ACT1* as a *CBK1*-interactor. Although *ACT1* is, of course, a crucial part of the cell cytoskeleton, it also plays an important role in activation of the cAMP/PKA pathway. The Sundstrom lab has shown that actin dynamics regulate PKA activity [44] and, recently, Zou *et al.* have elegantly demonstrated that actin functions as part of a PKA sensor/activator complex during hyphal development [45]. Indeed, decreased G-actin levels lead to decreased PKA pathway activity and, in turn, decreased filamentation in *C. albicans* [45]. As such, one explanation for the interaction between *ACT1* and *CBK1* is that the lowered *ACT1* gene dosage in the *act1*Δ/*ACT1 cbk1*Δ/*CBK1* mutant exacerbates the filamentation defects of decreased RAM pathway activity by concomitantly limiting PKA activity. This explanation also implies that the PKA pathway may compensate for decreased RAM pathway activity during morphogenesis.

### RAM pathway mutants show evidence of increased PKA pathway activity

**Figure 5:**
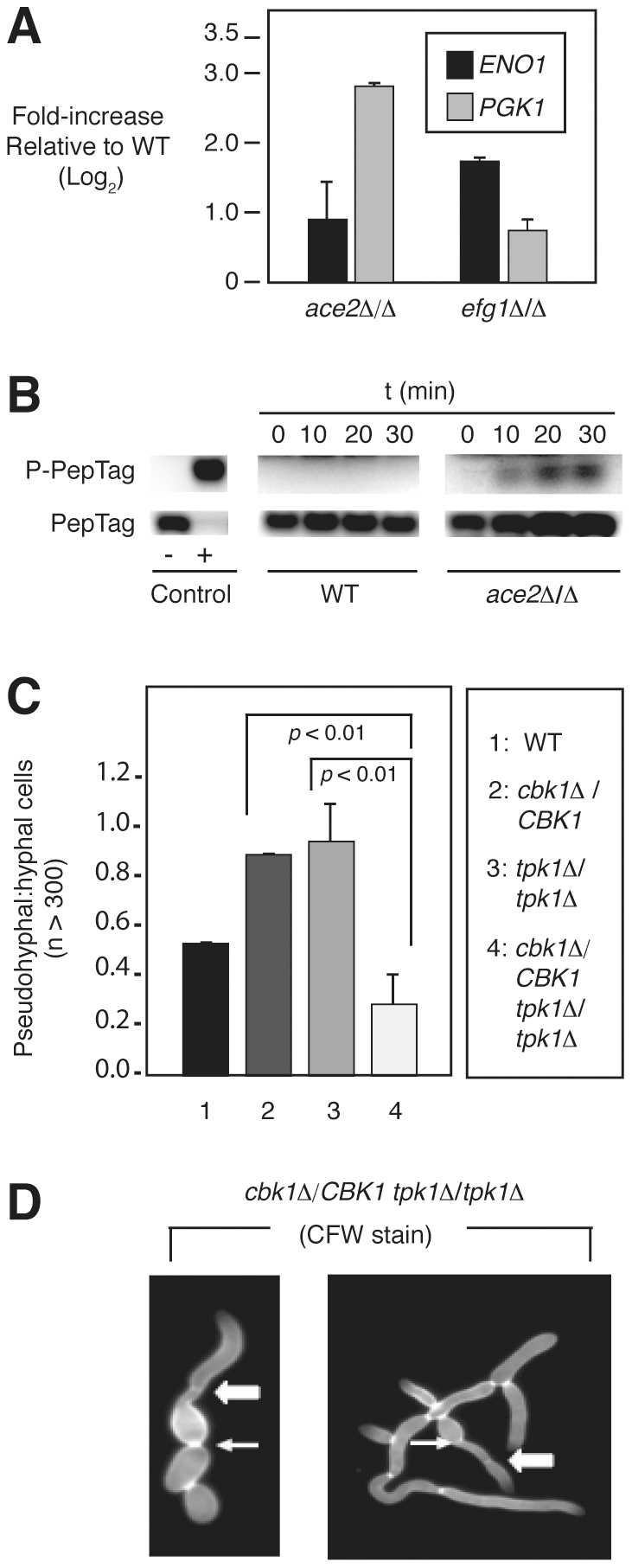
The PKA pathway compensates for decreased RAM pathway activity during morphogenesis. (A) Transcript levels of *ENO1* and *PGK1* in each mutant were compared to wild type by qRT-PCR using the 2^−*ΔΔCt*^ method and are graphed as Log_2_ change over wild type (three independent experiments performed in triplicate). Bars indicate mean value and error bars indicate standard deviation. The observed elevation in levels of each transcript in the indicated mutants relative to wild type were statistically significant by Student's *t* test (*p*<0.05). (B) Phosphorylation of fluorescent PKA substrate (PepTag, Promega) by cell extracts (10 µg protein) derived from wild type (WT) and *ace2*Δ/*2*Δ cells harvested after incubation in SM for 3 h at 37°C. The indicated time points represent PKA reaction time. (C) The ratio of pseudohyphal∶hyphal cells for the indicated strains was determined by light microscopy after 3 h incubation in liquid SM at 37°C. The bars indicate mean values of two-three independent replicates of at least 100 cells. Error bars indicate standard deviation. Brackets indicate the results of Student's *t* test evaluation of differences between the indicated mutants; *p*<0.05 indicates a statistically significant difference. (D) Hybrid pseudohyphae/hyphae cells of *cbk1*Δ/*CBK1 tpk1*Δ/*1*Δ following staining with Calcofluor white. Arrows indicate budneck localized septa (pseudohyphae-like) and block arrows indicate distal septa (hyphae-like).

To test the hypothesis that the RAM and PKA pathways regulate a common set of genes during morphogenesis, we examined the expression of two *CBK1*-interacting genes containing both Ace2 and Efg1 binding sites in *ace2*Δ/Δ and *efg1*Δ/Δ mutants after 3 hours of hyphal induction with SM. As shown in Figure 5A, the expression of the transcripts increased in both strains relative to wild type by quantitative RT-PCR. These observations suggest either that Efg1 and Ace2 are functioning as transcriptional repressors or that compensatory responses are occurring to maintain expression of these genes during morphogenesis when one of the two pathways is disabled.

To test the latter hypothesis, total cell lysates of the RAM pathway mutant *ace2*Δ/Δ were prepared and the level of PKA enzymatic activity determined after 3 hours exposure to hypha-inducing conditions (Figure 5B). At this time point, PKA activity has reduced to low levels in wild type cells [45], but there is clearly increased PKA activity in the *ace2*Δ/Δ mutant. This suggests that the PKA pathway is hyperactive in RAM pathway mutants and is consistent with the hypothesis that the PKA pathway may compensate for decreased RAM pathway activity. To further test the interaction between the RAM and PKA pathways, we deleted one allele of *CBK1* in strains containing homozygous null mutations in one of the catalytic subunits of the PKA enzyme [46] to yield the mutants *cbk1*Δ/*CBK1 tpk1*Δ/Δ and *cbk1*Δ/*CBK1 tpk2*Δ/Δ. The two triple mutants along with wild type and the parental mutants were incubated in SM for 3 hours at 37°C to induce filamentation. As shown in Figure 5C, deletion of *TPK1* in the *cbk1*Δ/*CBK1* background decreases the proportion of pseudohyphae formed by the *cbk1*Δ/*CBK1* mutant, while deletion of *TPK2* has no effect (data not shown), suggesting that the increased proportion of pseudohyphae formed by *cbk1*Δ/*CBK1* is dependent on *TPK1*. The phenotypic differences evident upon deleting the two isoforms of PKA are consistent with previous data indicating that they have distinct and redundant roles in filamentation [46].

Interestingly, cultures of *cbk1*Δ/*CBK1 tpk1*Δ/Δ in SM contained significant numbers of filaments that showed characteristics of both pseudohyphae and hyphae (Figure 5D). This hybrid morphology was not observed in cultures of wild type, *cbk1*Δ/*CBK1*, or *tpk1*Δ/Δ cells. Similar hyphae-pseudohyphae hybrid morphologies were recently observed by Carlisle *et al.* in cells expressing an intermediate level of *UME6* [47], suggesting that concurrent disruption of both RAM and PKA pathways interferes with the ability of the cell to commit to one morphotype. These observations also suggest that a balance between the activities of the PKA and RAM pathway is required for normal morphogenesis.

**Figure 6:**
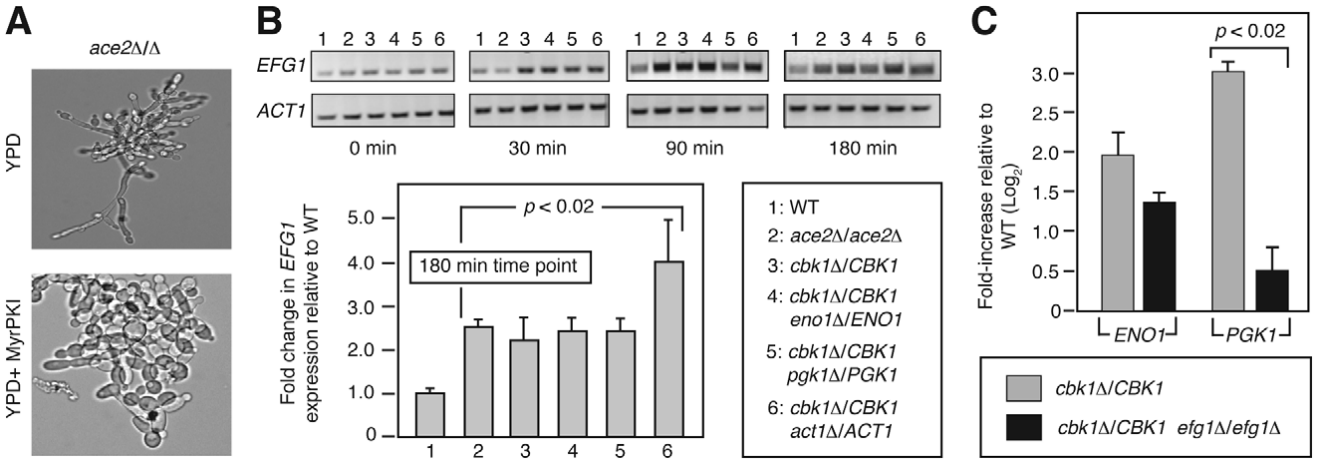
Elevated PKA activity accounts for increase pseudohyphae in RAM mutants. (A) *ace2*Δ/Δ cells were incubated in YPD at 30°C for 3 h −/+ PKA inhibitor MyrPKI (10 µM) and examined by light microscopy. (B) *EFG1* expression was determined by semi-quantitative RT-PCR for each strain at the indicated time after transfer to SM at 37°C. *ACT1* levels were used as loading control. The graph indicates the fold change in *EFG1* levels for the mutant strains relative to wild type at the 180 min time point. The bars indicate the mean fold change in *EFG1* relative to wild type for three independent replicates and the error bars indicate standard deviation. The brackets indicate that the difference between *EFG1* transcript levels was statistically significant for each mutant relative to wild type (Student's *t* test, *p*<0.02). (C) The expression of *ENO1* and *PGK1* were examined in the indicated strains as described in Figure 5A. The brackets indicate that the difference between *PGK1* transcript levels was statistically significant for the two mutants (Student's *t* test, *p*<0.02).

Increased and/or dysregulated PKA pathway activity has been linked previously to increased pseudohyphae formation. For example, Tebarth *et al.* have shown that overexpression of *EFG1* induces constitutive pseudohyphae [48]. We, therefore, hypothesized that elevated PKA activity might be responsible for the constitutively pseudohyphal phenotype displayed by *ace2*Δ/Δ as well as the increased proportion of pseudohyphae observed with *cbk1*Δ/*CBK1* heterozygotes showing CHI. Three observations support this hypothesis. First, treatment of *ace2*Δ/Δ cells with the substrate-based PKA inhibitor MyrPKI [49], under non-inducing conditions, significantly increased the number of yeast-like cells and decreased the number of mature pseudohyphae (Figure 6A), strongly supporting the notion that increased PKA activity is involved in the constitutive pseudohyphal phenotype of *ace2*Δ/Δ. Second, *EFG1* expression is elevated in both RAM pathway mutants and *cbk1*Δ heterozygotes relative to wild type over the time course of hyphal induction (Figure 6B). Densitometric analysis of three replicates of the 180 min time point indicates that the *EFG1* levels are 2–4 fold higher in each of the mutants relative to wild type (*p*<0.02, Student's *t* test). To further confirm this elevation, we compared the levels of *EFG1* in wild type and the double heterozygote *cbk1*Δ/*CBK1 pgk1*Δ/*PGK1*. Consistent with the semi-quantitative data, *EFG1* is elevated in *cbk1*Δ/*CBK1 pgk1*Δ/*PGK1* relative to wild type (4.8 log_2_, std. dev. 0.9, *p* = 0.01, Student's *t* test). Third, deletion of both alleles of *EFG1* in the *cbk1*Δ/*CBK1* background decreases expression of *ENO1* by a modest 1.5-fold and *PGK1* a more significant 8-fold relative to the parental strain (Figure 6C), indicating that at least a portion of the increased expression of putatively co-regulated genes in RAM mutants is mediated by the PKA-Efg1 pathway. Taken together, these experiments suggest that some of the *CBK1*-interacting genes isolated in our screen are part of the transcriptional output of both the PKA and RAM pathways and that decreased RAM function in the *CBK1* double heterozygotes leads to a compensatory increase in PKA pathway activity which, in turn, manifests as a phenotype of increased pseudohyphal growth due to increased *EFG1* levels [45].

### A balance between RAM and PKA pathwzay activity is required for normal morphogenesis

**Figure 7:**
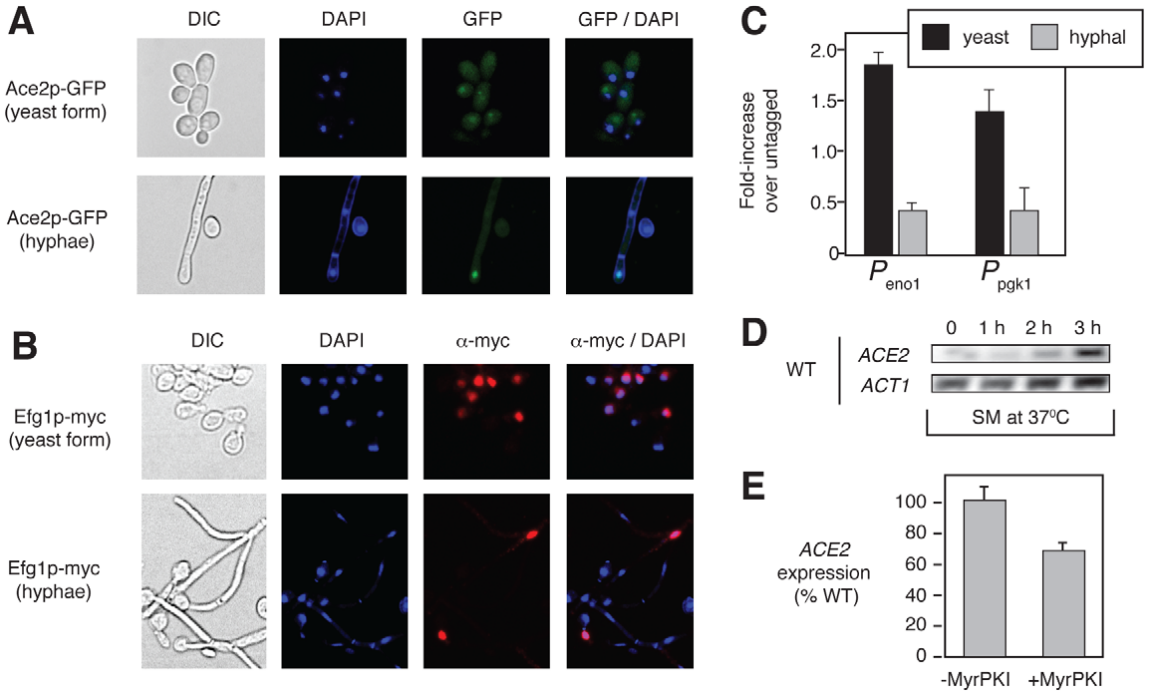
Efg1 and Ace2 are present in the nuclei at different time points during morphogenesis. (A) The localization of Ace2-GFP was determined in stationary phase cells prior to initiation of hyphal induction (yeast form) and after 3 hr hyphal induction in SM. DAPI staining was used to identify the nuclei. (B) The localization of Efg1-Myc was determined by indirect immunofluorescence under conditions identical to those described for Ace2-GFP. (C) The binding of Efg1-Myc to promoter regions of *ENO1* and *PGK1* was examined by ChIP for cells corresponding to the time points examined in A and B. (*D*) *ACE2* expression in wild type (WT) cells compared to *ACT1* by RT-PCR at the indicated times after hyphal induction in SM at 37°C. (*E*) *ACE2* expression was determined in wild type cells −/+ PKA inhibitor (MyrPKI, 10 µM) after 3 h induction in SM at 37°C.

Although our results strongly suggest that the RAM and PKA pathways interact during morphogenesis and that the PKA pathway may be hyper-activated in the absence of RAM activity, it remained to be determined how these pathways interact during normal morphogenesis. As discussed above, one of the best characterized functions of Ace2 in both *S. cerevisiae* and *C. albicans* is as a daughter cell-specific transcription factor [26], [38], [39]. Two other laboratories [26], [39] have previously shown that in *C. albicans*, Ace2 localizes to daughter nuclei in actively dividing yeast-phase cells as well as in serum-induced filaments; our results confirm those findings in SM (Figure 7A). We, therefore, hypothesized that the relative contributions of Ace2 and Efg1 to gene regulation during the course of hyphal development may correspond to the timing of their nuclear localization. To our knowledge, the nuclear localization of Efg1 during filamentation had not been described previously.

To test this hypothesis, we used indirect immunofluorescence to compare the proportion of cells with nuclear Efg1 at the initiation of hyphal development to the proportion in hyphal cell nuclei. As shown in Figure 7B, Efg1 is present in the nuclei of 50–60% (n = 100 cells) of cells prior to shifting to SM. In contrast, Efg1 is detectable in only ∼10% of hyphal nuclei. Correspondingly, Efg1 occupancy of the promoter regions of *ENO1* and *PGK1* is also higher at the initiation of hyphal development by ChIP analysis (Figure 7C). This suggests that Efg1 may be more important at the onset of, or early in, the filamentous transition, while Ace2 contributes to Efg1/Ace2 co-regulated gene transcription as daughter cell nuclei accumulate within the hyphal structure.

Consistent with this model, *ACE2* expression increases over the 3-hour time course of hyphal induction (Figure 7D); this finding is also consistent with its role in gene expression within daughter cell nuclei. Interestingly, the promoter region of *ACE2* has five Efg1 consensus binding sites, suggesting that the PKA pathway may contribute to the regulation of *ACE2* expression. However, treatment with the PKA inhibitor MyrPKI reduced levels of *ACE2* expression only modestly after 3 hours in SM (Figure 7E). Although this observation supports a possible direct link between the PKA and RAM pathways, it suggests that PKA-Efg1 is not the sole, or even dominant, regulator of *ACE2* expression.

As a whole, these data support a model in which Efg1 plays a more important role at the initiation of hyphal development in SM, and Ace2 plays a more important role once daughter nuclei accumulate within the hyphal structure. Since *EFG1* expression is maintained throughout the time course of hyphal development (Figure 6B) and Efg1 is present in some hyphal nuclei (Figure 7A), it is unlikely that the relationship between Ace2 and Efg1 represents an “either/or” type of scenario. Instead, it seems more likely that a balance exists between the relative contributions of the two transcription factors to gene expression and that this balance varies during hyphal development.

## Discussion

Methods for the large-scale genetic analysis of *Candida albicans* have advanced tremendously in recent years, leading to a number of important and informative studies [14]–[20]. To our knowledge, however, no large-scale synthetic genetic analyses have yet been reported. Here, we present the first such screen. Our approach was based on a CHI strategy, and, like other large-scale genetic analyses of *C. albicans*, we employed transponson-mediated insertional mutagenesis to generate a large collection of double heterozygous mutants derived from a parental strain containing a heterozygous null mutation of the RAM pathway kinase *CBK1*. This library was then used to screen for genes that interacted with *CBK1* during SM-induced morphogenesis.

First and foremost, our data establishes that CHI-based genetic interaction screening is a useful method for the genetic analysis of the obligate diploid yeast *C. albicans*. A priori, CHI-based genetic screening of a signaling network such as the RAM pathway would be expected to identify genes that interact with the query gene through a variety of mechanisms. Inspection of our dataset confirms these expectations in that it includes transcriptional targets of the RAM pathway (e.g., *ENO1*, *PGK1*), genes that likely affect the function of pathway components (e.g., *NSP1*, *SLK19*), and genes that function in parallel pathways (e.g., *MAF1*, *SLF1*). In the specific case of screening a protein kinase mutant, it should also be possible to identify substrates of that kinase. Although no bona fide substrate of Cbk1 has been confirmed in *C. albicans*, our screen identified a very likely candidate in Ssd1. Ssd1 is a well characterized Cbk1 substrate in *S. cerevisiae* [34] and has been shown previously to interact genetically with *CBK1* in both *S. cerevisiae* [35] and *C. albicans* [27]. A consensus Cbk1 phosphorylation sequence has recently been identified in *S. cerevisiae* [34]. Supporting the possibility that *Ca*Ssd1 is a substrate of *Ca*Cbk1 is the presence of this consensus phosphorylation sequence. Of the remaining *CBK1*-interactors, *RGD3*, an uncharacterized potential Rho GTPase, and *VPS13*, a protein involved in vacuolar protein sorting, also have sequences that match the consensus phosphorylation sequence for *Sc*Cbk1 (data not shown). Studies directed towards confirming these putative Cbk1 substrates are in progress.

The *CBK1*-derived double heterozygous mutants isolated in our screen displayed phenotypes indicative of defects in the Ace2-dependent functions of the RAM pathway in that they were only observed on SM [25]; mutations in genes affecting Ace2-independent functions would be expected to display filamentation defects on both SM and serum [27]. Since many of the interacting genes appear to be transcriptional targets of Ace2, we propose that the effect of partially disabling the RAM pathway by deletion of one allele of *CBK1* is exacerbated by further deletion of one allele of a gene regulated by the *CBK1*-dependent transcription factor Ace2. The cumulative effect of these two mutations results in phenotypes (increased proportion of pseudohyphae) consistent with a further decrease in Ace2-mediated RAM transcriptional activity. By this analysis, Ace2-transcriptional targets that display CHI interactions with *CBK1* would, therefore, appear to be particularly important components of the transcriptional output of the RAM pathway during morphogenesis on SM.

A particularly powerful feature of synthetic genetic analysis is the ability to identify interactions between regulatory pathways and, in this regard, our CHI screen of *cbk1*Δ/*CBK1* was quite informative, highlighting the interplay between the RAM and PKA pathways during morphogenesis. Although no components of the PKA signaling pathway were identified as *CBK1*-interactors, analysis of the dataset revealed that many of the interactors were regulated by the PKA pathway. Indeed, the similar transcriptional characteristics of the PKA-regulated transcription factor Efg1 and Ace2 in *C. albicans* have been previously noted [50] and, while our work was in progress, Wang *et al.* reported that Efg1 and Ace2 bound to the promoters of *C. albicans* genes involved in cell separation [39]. In addition, the PKA and RAM pathways have been linked genetically in *S. cerevisiae* through experiments showing that ectopic over-expression of the PKA kinase subunit *TPK1* suppresses growth and budding defects of RAM pathway mutants in an Ace2-*independent* manner [51]. Our data suggest that the PKA and RAM pathway interact in *C. albicans* with respect to Ace2-dependent functions.

Consistent with this model, consensus binding sites for both Efg1 and Ace2 are located in the promoter regions of a significant proportion of *CBK1*-interactors. A genome-wide search identified 384 putative Efg1/Ace2 co-regulated genes, suggesting that the two pathways interact to modulate the expression of a substantial subset of genes. The interaction of these two pathways is further supported by our isolation of two PKA-regulated transcriptional modulators (*MAF1* & *SLF1*) as *CBK1* interactors as well as by the synthetic genetic interactions between *CBK1* and *TPK1* observed in our follow-up studies.

The simplest manifestation of a model in which the PKA and RAM pathways co-regulate a set of genes would be that deletion of either *ACE2* or *EFG1* results in the decreased expression of co-regulated genes. As shown in Figure 5A, this is not the case as the expression of putatively co-regulated genes is increased in both *ace2*Δ/Δ and *efg1*Δ/Δ mutants. This suggested that the two pathways may compensate for one another when the other pathway is disabled. Supporting this notion, the activity of the PKA pathway is increased in RAM pathway mutants (Figure 5B), and *EFG1* mediates a substantial portion of the increased expression of co-regulated genes in the absence of full RAM pathway activity (Figure 6B). Accordingly, the level of *EFG1* expression is also increased (Figure 6B) and, since inappropriately high levels of *EFG1* promote pseudohyphal growth (48), this observation provides an explanation for the increased amounts of pseudohyphae displayed by RAM pathway mutants.

We, therefore, propose that the increased PKA activity in RAM pathway mutants represents a compensatory response that maintains expression of Ace2/Efg1 co-regulated genes in the absence of a fully functional RAM pathway. However, constitutively elevated levels of PKA activity represent a dysregulated state and, consequently, the expression levels of the genes are not returned to normal but are elevated. Thus, it appears that a balance between the activity of the PKA and RAM pathways is required to maintain properly regulated expression of co-regulated genes. Maintaining a balance between the activities of the two pathways appears to be required for normal hyphal development because: 1) loss of *EFG1* leads to a failure to form filaments; 2) loss of *ACE2* leads to the accumulation of pseudohyphae; and 3) concurrent partial disruption of both pathways leads to the formation of filaments with characteristics of both hyphae and pseudohyphae (Figure 5D).

If, as our results suggest, a balance between PKA and RAM pathway-mediated transcription is required for the cell to normally undergo filamentation, then how is this balance established and maintained? Although further work will be required to determine the molecular mechanism of this interaction, the cell cycle-regulated nature of both Efg1 and Ace2 suggests that the pathways might be active at different times during morphogenesis. Ace2, for example, localizes to the nuclei of daughter cells in both yeast and filamentous *C. albicans* [26], [39]. Efg1, on the other hand, has been shown to be rapidly down-regulated soon after hyphal induction in some conditions [48]. These considerations led us to propose that Efg1 may be more important in the expression of co-regulated genes earlier in morphogenesis, while Ace2 is the dominant regulator later in morphogenesis when daughter nuclei appear within the filament.

Consistent with that model, we showed that more nuclei contain Efg1 at the initiation of morphogenesis than later in the process. Ace2, on the other hand, is absent from the vast majority of nuclei at the initiation of morphogenesis but is found in daughter nuclei as they accumulate within the filament (Figure 7A). Consistent with its role later in morphogenesis, overall expression of *ACE2* also increases as the cells are exposed to inducing condition for longer periods of time (Figure 7D). Since Efg1 remains detectable in hyphal nuclei (Figure 7B), it is unlikely that Ace2 replaces Efg1 entirely but rather Ace2 may become relatively more important as daughter nuclei accumulate within the filament and undergo mitosis. Thus, it seems that a balance between the PKA and RAM pathways exists and that this balance is important for smooth morphogenesis. A potential illustration of the importance of this balance is provided by the morphologies displayed by the *tpk1*Δ/Δ *cbk1*Δ/*CBK1* mutant in which single filaments show characteristics of both hyphae and pseudohyphae.

This model is also consistent with the observations of Wang *et al.*, who reported that Efg1 represses the expression of Ace2-regulated cell separation genes during hyphal development [39]. They found that in wild type strains, the Ace2-regulated expression of chitinase *CHT3* occurs approximately 3 hours post-hyphal induction, a point at which multiple septa and daughter nuclei have formed within the hyphal filament. The 3-hour time point also corresponds to the time when we observed high levels of *ACE2* expression. In *EFG1* mutants, on the other hand, Wang *et al.* found that *CHT3* is inappropriately expressed within the first hour of induction and is expressed at higher levels at 3 hours [39]. Our observations regarding the timing of Efg1 nuclear localization correlate well with these expression data in that Efg1 is present early when it suppresses Ace2-mediated *CHT3* expression but is absent when *CHT3* expression is induced. It is important to note that Efg1 has previously been proposed to function as both a transcriptional activator and repressor during hyphal morphogenesis [39], [41] and, taken together with the observations of Wang *et al.*, our data are consistent with such a role.

At this point, further work will be required to understand the molecular mechanisms by which the RAM and PKA pathway interact. As noted above, *ACE2* does possess a number of Efg1 consensus binding sites within its promoter. This suggests a possible feed-forward mechanism by which Efg1 activates the expression of *ACE2*, which, in turn, takes over transcription of co-regulated genes. However, chemical inhibition of the PKA pathway only modestly reduced expression of *ACE2* during hyphal induction (Figure 7E). Similarly, *efg1*Δ/Δ mutants also exhibit very slight changes in *ACE2* expression (data not shown). Although there may be an operative component of this feed-forward mechanism, it seems to be a relatively minor contributor to the crosstalk between these pathways.

In summary, we have shown that CHI-based genetic interaction screening is a useful approach for the analysis of complex phenotypes in *C. albicans*. The application of this approach to the RAM pathway has provided insights into the mechanisms by which the PKA and RAM signaling pathways function together during the transition from yeast to filamentous cells in *C. albicans*.

## Materials and Methods

### Strains, media, and growth conditions

All strains are derived from CAI4 (*ura3*Δ::*imm*434/*ura3*Δ::*imm*434). CAMM-292 (*ura3*Δ::*imm*434/*ura3*Δ::*imm*434/*cbk1*-Δ1::*his*G/*CBK1*) [24] was used as the parental strain for transposon mutagenesis. A complete list of strains and genotypes is provided in Table S1. Yeast peptone dextrose supplemented with 80 mg/L uridine, synthetic dextrose medium lacking uracil, and SM were prepared using standard recipes [15], [52]. Induction of filamentation was carried out using SM plates (37°C, 3D) or liquid SM (37°C, 3 h). All phenotypes were confirmed on SM plates supplemented with uracil to control for possible positional effects of *URA3* expression. Proportions of yeast, pseudohyphae and hyphae in liquid cultures were determined by light microscopy using morphological scoring criteria described by Sudbery *et al.* [53].

### Transposon mutagenesis

*C.albicans* strain WO-1 pEMBLY23 genomic DNA library (NIH AIDS Research & Reference Reagent Program) was mutagenized (9 independent reactions) *in vitro* using the GPS3-Mutagenesis system from New England Biolabs (Beverly, MA) and a donor plasmid (pGPS3) containing the *CaURA3-dpl200* cassette [16] inserted at the *Spe* I restriction site. Mutagenized genomic fragments were released by PvuII digestion and transformed into CAMM-292 using a lithium acetate-protocol with heat shock at 44°C for 20 min [54]. The library is available upon request from the Kumar laboratory (akumar@umich.edu).

### Identification of transposon insertion sites

Transposon insertion sites were amplified by 3′ RACE (rapid amplification of cDNA ends) using primers complementary to the ends of the transposon construct, cloned into a TA vector, and sequenced. Insertion sites were then identified by BLASTN searches using the Candida Genome Database (www.candidagenome.org).

### Construction of *cbk1*Δ/*CBK1*-derived double heterozygotes

Ten double heterozygotes that showed CHI were independently constructed from the Ura- parental strain *cbk1*Δ/*CBK1* (CAMM292) using fusion PCR methods to generate *URA3*-based knockout cassettes [33]. The cassettes were used to transform CAMM292 to Ura prototrophy, and correct integration was confirmed by PCR. Two independent isolates were evaluated for all phenotypes.

### qRT-PCR and Chromatin Immunoprecipitation assays

Total RNA was isolated using the RiboPure Yeast Kit (Ambion, Austin, TX) and reverse transcribed using the SuperScript III First Strand Synthesis Kit (Invitrogen, Carlsbad, CA). Changes in transcript levels of target genes were analyzed using the Platinum SYBR Green Mix (Invitrogen) and normalized to *ACT1* levels using the 2^−ΔΔ*Ct*^ method [55]. ChIP assays were performed as described previously [56] using Ura+ CAI4-dervatives containing *ACE2*-TAP and *EFG1*-MYC alleles.

### Protein kinase A assay

Protein kinase A activity was measured in total cell lysates using the PepTag cAMP-dependent protein kinase assay kit (Promega, Madison WI) following a protocol previously developed for *C. albicans* [57]. Lysates were prepared from wild type and *ace2*Δ/Δ cells that had been exposed to SM for 3 h. Phosphorylation of the PepTag substrate was determined by agarose gel electrophoresis; the unphosphorylated substrate migrates in the opposite direction as the phosphorylated substrate. Images of the gel were captured on a gel-doc imaging system and processed using Adobe PhotoShop software. Identical contrast and levels were used for each image.

### Microscopy

Light and fluorescence microscopy was performed using a Nikon ES80 epi-fluorescence microscope equipped with a CoolSnap CCD camera. Images were collected using NIS-Elements Software and processed in PhotoShop. Indirect immunofluorescence was performed as previously described using anti-Myc (Invitrogen) primary- and TexasRed-conjugated (Molecular Probes) secondary- antibodies [58]. DAPI and Calcofluor white staining was performed as described [52].

## Supporting Information

Table S1 Strains.
(DOC)

Table S2 Table of GO terms, number of genes per GO category, p-values and example ORFs containing both Ace2 (MMCCASC) and Efg1 (CANNTG) binding sites within 1000 bp of the start codon. ORFs were identified by searching the CGD database (www.candidagenome.org) and analyzed using GO toolbox statistical software (http://genome.crg.es/GOToolBox/).
(DOC)
