# Supplementary material for: A Large-Scale Complex Haploinsufficiency-Based Genetic Interaction Screen in Candida albicans: Analysis of the RAM Network during Morphogenesis
Source: PLoS Genet. 2011 Apr 28;7(4):e1002058. doi: 10.1371/journal.pgen.1002058 (PMC3084211; doi:10.1371/journal.pgen.1002058)
Supplement: Table S1 — Strains. (DOC) [file pgen.1002058.s001.doc]

**Table S1. Strains**

| **Strain** | **Genotype** | **Reference** |
| --- | --- | --- |
| CAI4 | *ura3∆::λimm434*/*ura*3*∆::λimm434* | **1** |
| CAF2 | *URA3*/*ura*3∆*::λimm434* | **1** |
| CAMM29 | as CAI4 with *cbk1::URA3/CBK1* | **2** |
| CAM292 | as CAMM29 with *cbk1*::*λimm434/CBK1* | **2** |
| MK62 | *ura3∆::λimm434*/*ura*3*∆::λimm434his1::hisG/his1::hisGace2∆::HIS1/ace2∆::URA3* | **3** |
| HLC52 | as CAI4 with *efg1∆::hisG/efg1∆::hisG-URA3-hisG* | **4** |
| YCRY1 | as CAM292 with *cbk1*::*λimm434/cbk1::URA3* | **This work** |
| YCRY2 | as HLC52 with *cbk1::URA3/CBK1* | **This work** |
| IIHH6-4a | as CAI4 with *tpk1*∆::*hisG/tpk1∆::hisG* | **5** |
| SSY1 | as IIHH6-4a with *cbk1::URA3/CBK1* | **This work** |
| *ACE2-TAP* | As CAI4 with *ace2::TAP::URA3/ACE2* | **This work** |
| *ACE2-GFP* | BWP17, ace2::*ACE2*-GFP-*URA3*/ace2::*ACE2-GFP-HIS1* | **Gift of**  **C. Vasquez** |
| HLY3499 | As CAI4 *efg1::hisG/EGF1-MYC-URA3* | **6** |

1. Fonzi WA, Irwin MY (1993) Isogenic strain construction and gene mapping in *Candida albicans*. Genetics 134:717-728
2. McNemar MD, Fonzi WA (2002) Conserved serine/threonine kinase encoded by *CBK1* regulates expression of several hypha-associated transcripts and genes ecoding cell wall proteins in *Candida albicans*. J Bacteriol 184:2058-2061.
3. Kelly MT, MacCallum DM, Clancy SD, Odds FC, Brown AJ, et al. (2004) The *Candida albicans* Ca*ACE2* gene affects morphogenesis, adherence, and virulence. Mol Microbiol 53:969-983.
4. Lo HJ, Kohler JR, DiDomenico B, Loebenber D, Cacciapuoti A, et al. (1997) Nonfilamentous *C. albicans* mutants are avirulent. Cell 90:939-949.
5. Bockmuhl DP, Krishnamurthy S, Gerads M, Sonnedborn A, Ernst JF (2001) Distinct and redundant froles of the two kinase A isoforms Tpk1p and Tpk2p in morphogenesis and growth of *Candida albicans*. Mol Microbiol 42:1243-1257.
6. Wang A, Raniga PP, Lane S, Lu Y, Liu H. (2009) Hyphal chain formation in *Candida albicans*; Cdc28-Hgc1 phosphorylation of Efg1 represses cell separation genes. Mol Cell Biol 29:4406-4416.
