# Supplementary material for: A Large-Scale Complex Haploinsufficiency-Based Genetic Interaction Screen in Candida albicans: Analysis of the RAM Network during Morphogenesis
Source: PLoS Genet. 2011 Apr 28;7(4):e1002058. doi: 10.1371/journal.pgen.1002058 (PMC3084211; doi:10.1371/journal.pgen.1002058)
Supplement: Table S2 — Table of GO terms, number of genes per GO category, p-values and example ORFs containing both Ace2 (MMCCASC) and Efg1 (CANNTG) binding sites within 1000 bp of the start codon. ORFs were identified by searching the CGD database (www.candidagenome.org) and analyzed using GO toolbox statistical software (http://genome.crg.es/GOToolBox/). (DOC) [file pgen.1002058.s002.doc]

**Table S2**.
